# Tianma Gouteng Decoction Exerts Cardiovascular Protection by Upregulating OPG and TRAIL in Spontaneously Hypertensive Rats

**DOI:** 10.1155/2020/3439191

**Published:** 2020-10-20

**Authors:** Lin-hua Deng, Lan Li, You Zhai, Sarhene Michael, Chun-yang Yang, Rui Guo, Shan-fu Chen, Guan-wei Fan, Ying-qiang Zhao, Wei Liu

**Affiliations:** ^1^Second Affiliated Hospital of Tianjin University of Traditional Chinese Medicine, Tianjin 300193, China; ^2^Tianjin University of Traditional Chinese Medicine, Tianjin, China; ^3^Tianjin Key Laboratory of Translational Research of TCM, Tianjin, China; ^4^First Teaching Hospital of Tianjin University of Traditional Chinese Medicine, Tianjin, China

## Abstract

Tianma Gouteng Decoction (TGD) is widely used in traditional Chinese medicine for the treatment of hypertension and its related complications, but its mechanisms remain incompletely defined. We now aim to assess the protective effect of TGD against cardiovascular damage and to investigate its characteristics and underlying mechanisms. Blood pressure was determined in TGD-treated spontaneously hypertensive rats (SHR) by noninvasive measurements. Echocardiography was performed to assess cardiac function and structure and sirius red staining to evaluate cardiac fibrosis, and the degree of vascular remodeling was evaluated. Additionally, vasoconstriction and relaxation factor expression changes were examined by means of ELISA. Protein expression changes were verified by western blot. Compared with untreated SHR, TGD-treated SHR exhibited cardiovascular traits more akin to those of the normotensive Wistar Kyoto (WKY) rats. That is, they had lower diastolic blood pressure, systolic blood pressure and mean BP, and increased expression of vasodilation factor. We also found that TGD reduces ventricular and vascular remodeling and improves cardiac function in SHR. Finally, we tested the antiapoptosis effect TGD exerts in SHR, ostensibly by upregulating the expression of OPG, TRAIL, and death receptor 5 (DR5) and downregulating caspases 8, 7, and 3. TRAIL may also exert antiapoptotic and prosurvival effects by upregulating AKT expression. Therefore, TGD may reverse cardiovascular remodeling in SHR by upregulating the expression of OPG and TRAIL, upregulating AKT, and inhibiting apoptosis, at least in part. For the first time, we have shown that OPG and TRAIL play complimentary cardioprotective roles in SHR.

## 1. Introduction

Hypertension is a major public health issue worldwide, but the rates of treatment and control are still very low [1]. In recent results from a 1,100,507 participant study in low- and middle-income countries, 192,441 (17.5%) had hypertension; among those with hypertension, 39.2% were newly diagnosed with hypertension and 29.9% of those with hypertension received treatment, but only 10.3% of participants achieved control of their hypertension [2]. Even in high-income countries, the control rate was still less than 25% [3]. Meanwhile, hypertension is also the major contributing factor in premature deaths in China. It is estimated that one-third of Chinese adults have high blood pressure, and the rate of hypertension control is only 13.0% [4]. Because asymptomatic involvement of multiple organs (including heart, brain, and kidney) in hypertensive patients is an independent determinant of cardiovascular risk [5], the burden of disease caused by hypertension in China is very serious. The pathogenesis of hypertension is complex, including some that are well known, such as activation of the Renin-Angiotensin-Aldosterone System (RAAS), abnormalities of the sympathetic nervous system, impaired endothelial function, and insulin resistance. In addition to high blood pressure, these also cause excessive fibroblast proliferation and increase the accumulation of collagen. Furthermore, hypertension stimulates cardiomyocyte apoptosis and inflammation, leading to the development of diffuse fibrosis and left ventricular hypertrophy, which accelerates heart remodeling. Therefore, finding the best intervention treatment strategy is a key issue.

Osteoprotegerin (TNFSF11B, OPG) is a soluble member of the TNF superfamily and was first described as an important regulator of osteoclast formation 20 years ago [6]. Recent studies have shown that OPG is also closely associated with angiogenesis [7]. There is much evidence that serum OPG is resistant to vascular calcification and is associated with a variety of disease processes, including dysmotility, atherosclerosis, and vascular calcification [8, 9]. At the same time, OPG is also a decoy receptor for TNF-related apoptosis-inducing ligands (tnfsf10, TRAIL, and Apo2L). By combining with TRAIL, OPG can inhibit the apoptosis of many different cell types induced by TRAIL, including those of smooth muscle cells [8], endothelial cells [10]. However, TRAIL can not only promote cell apoptosis but also activate antiapoptotic pathways. It has a protective effect in maintaining vascular homeostasis and antiatherosclerosis [11]. Study has shown that a lack of TRAIL increases vascular inflammation [12]. In addition, TRAIL promotes the survival and proliferation of primary human vascular endothelial cells by activating AKT and ERK pathways [13]. Based on the duality of TRAIL, the role of OPG/TRAIL system in cell apoptosis is worth exploring.

Tianma Gouteng Decoction (TGD) is made up of 11 commonly used prescriptions (*Uncaria*, *Gastrodia elata*, *Scutellaria baicalensis* Georgi., *Eucommia ulmoides* Oliv., achyranthes root, *Loranthus parasiticus*, abalone shell, Gardenia, *Leonurus japonicus*, caulis polygoni multiflori, and *Poria cocos*. Chinese pharmacopoeia (2015 edition) reported that, in recent decades, TDG has been widely used in the clinical treatment of symptoms caused by hypertension, such as dizziness and headache [14], TGD may be an effective treatment for essential hypertension with fewer side effects than western medicine. Furthermore, in a recent study, TGD was found to reduce the loss of dopaminergic neurons in rats with Parkinson's disease, to reduce the apoptosis of SH-SY5Y cells, and to exhibit additional neuroprotective effects [15]. TGD is also used clinically to effectively lower blood pressure. In our previous work, it was confirmed that gastrodin, the main component of *Gastrodia elata*, can effectively intervene in RAAS and PPAR*γ*, thereby exerting antihypertensive effect [16]. However, there is no definitive experimental evidence supporting the clinical application of TGD in the treatment of hypertension. In this study, we used spontaneously hypertensive rats (SHR) to study the antiapoptotic effect of TGD via OPG and TRAIL, thereby providing cardiovascular protection by exerting a hypotensive mechanism.

## 2. Materials and Methods

### 2.1. Materials

#### 2.1.1. Animals and Drug Treatment

Male spontaneously hypertensive rats (SHR) (*n* = 15) and Wistar Kyoto (WKY) (*n* = 5) rats (14 weeks old) were obtained from Vital River Laboratory Animal Technology Co., Ltd. (Beijing, China). All the rats were kept in a temperature-controlled room (22°C ± 2°C) with 40% ± 5% humidity, exposed to ordinary lighting (12 h light; 12 h darkness). The experimental procedures conformed to Directive 2010/63/EU of the European Parliament, and all animals were handled according to the guidelines of the TCM Animal Research Committee (TCM-LAEC2014005) of Tianjin University. SHR were randomly divided into three groups: (1) saline (*n* = 5, 2.5 mL/kg/d, 12 weeks, via i.g.), (2) TGD 20 g/kg/d (low dose, *n* = 5, 12 weeks, via i.g.), (3) TGD 40 g/kg/d (high dose, *n* = 5, 12 weeks, via i.g.). A group of WKY rats (saline, *n* = 5, 2.5 mL/kg/d, 12 weeks, via i.g.) was used as a control.

#### 2.1.2. Preparation of Tianma Gouteng Decoction

Tianma Gouteng Decoction from *Gastrodia elata* 10 g, *Uncaria* 15 g (later fried), abalone shell 20 g (first fried), Gardenia 6 g, *Scutellaria baicalensis* Georgi 6 g, achyranthes root 12 g, *Eucommia ulmoides* Oliv. 12 g, *Leonurus japonicus* 12 g, *Loranthus parasiticus* 12 g, dried caulis of polygoni multiflori 12 g, and *Poria cocos* 12 g. All of the above ingredients were provided by the Second Affiliated Hospital of Tianjin University of Traditional Chinese Medicine. TGD is concentrated into granules; each 1 g granule is equal to 10 g of raw materials and complies with the guidelines of Good Manufacturing Practices and Good Laboratory Practices formulated by Chinese government agencies. The TGD was dissolved in pure water to prepare solutions having a concentration of 200 mg/mL or 400 mg/mL for the experiment.

Saline was purchased from China Otsuka Pharmaceutical Co., Ltd. (Tianjin, China). The enzyme-linked immunosorbent assay (ELISA) kits, including prostacyclin (PGI2), thromboxane A2 (TXA2), angiotensin II (Ang II), and endothelin 1 (EDN1), were all obtained from Uscn Life Science, Inc. (Wuhan, China). The primary antibodies of GAPDH, AKT, p-AKT, caspase 8, caspase 7, caspase 3, and secondary antibodies were purchased from Proteintech Inc. (IL, USA). The antibodies of OPG, TRAIL, and DR5 were purchased from Abcam PLC (Cambridge, UK).

### 2.2. Methods

#### 2.2.1. Monitoring of Blood Pressure, Heart Rate, and Weight

The blood pressure, heart rate, and bodyweight of the rats were monitored weekly on conscious animals by a noninvasive tail sleeve method using an animal sphygmomanometer (BP98AWU, Softron Co., Ltd., Tokyo, Japan). For each rat, blood pressure and heart rate were measured by multiple readings until 5 stable measurements were obtained continuously. The data were calculated as the average blood pressure and heart rate values. A conventional hundredth gram scale was used for weighing every week.

#### 2.2.2. Serum Enzyme-Linked Immunosorbent Assay

The serum levels of PGI2, TXA2, EDN1, and Ang II were determined using the ELISA kits according to the manufacturer's protocol.

#### 2.2.3. Echocardiographic Assessment of Left Ventricular Function and Structure

All rats were examined by echocardiography at 12 weeks after initial drug administration. A Vevo 2100 (VisualSonics, Toronto, Canada) ultra-high-resolution animal ultrasound imaging system was used to evaluate left ventricle function and structure, including systolic interventricular septum (IVSs), diastolic interventricular septum (IVSd), systolic left ventricular posterior wall (LVPWs), diastolic left ventricular posterior wall (LVPWd), ejection fraction (EF%), and left ventricular mass (LVmass).

#### 2.2.4. Histopathology of the Aorta

After induction of anesthesia by intraperitoneal injection of 5% chloral hydrate (300 mg/kg), euthanasia was performed by excessive inhalation of isoflurane. The aorta was removed and fixed in a 4% paraformaldehyde solution for more than 48 hours, and then further prepared for paraffin sectioning. Serial sections (5 *μ*m) were excised and stained with hematoxylin-eosin. The sections were analyzed under a light microscope. Images were captured by a digital camera connected to an optical microscope (Digital Sight Leica DM3000, Leica Microsystems, Wetzlar, Germany) and processed using the Leica Application Suite (Leica Application Systems, Leica Application Suite, Wetzlar, Germany).

#### 2.2.5. Histopathology of the Heart

Similarly, the heart was dissected out and fixed in 4% paraformaldehyde solution for more than 48 hours, then further prepared for paraffin sectioning, cut into serial sections (5 *μ*m), and stained with sirius red. Images were captured by a digital camera connected to an optical microscope (Digital Sight Leica DM3000, Leica Microsystems, Wetzlar, Germany) and processed using the Leica Application Suite (Leica Application Systems, Leica Application Suite, Wetzlar, Germany). To quantify the fibrosis, we used the image analysis system ImageJ (US National Institutes of Health, Bethesda, MD, USA) to calculate the proportion of red stained area within the frame.

#### 2.2.6. Western Blot Analysis

The rats were sacrificed under anesthesia, and about 50 mg of myocardial tissue was taken out from the left ventricle and stored at −80°C (*n* = 3). The whole left ventricle protein was extracted and subjected to radioimmunoprecipitation assay (RIPA) lysis buffer (Sangon Biotech Co., Ltd., Shanghai, China). The total protein concentration was determined using a bicinchoninic acid (BCA) protein assay kit (Tianjian Biotechnology (Beijing) Co., Ltd., Beijing, China) according to the instructions, and the average value of the concentrations was calculated. The sample was then stored at −80°C. Total protein was mixed with 3 *μ*L of 5x SDS sample buffer. After separation by sodium dodecyl sulfate-polyacrylamide gel electrophoresis (SDS-PAGE) (12% gel), the protein was transferred to a polyvinylidene fluoride membrane (PVDF membrane). After blocking with 5% skim milk powder or 5% bovine serum albumin (BSA) for 2 h, it was washed 4 times with Tris-HCl buffered saline (TBS) Tween for 7 min, and the membrane with the target protein was cut for incubation. The primary antibodies used were OPG, TRAIL, procaspase 8, cleaved caspase 8, caspase 7, procaspase 3, cleaved caspase 3, AKT, and p-AKT. All antibodies were diluted according to the manufacturer's instructions and left overnight at 4°C. Then, the membrane was washed 3 times for 5 min each and incubated with the secondary antibody for 2 h at room temperature and then washed 5 times with TBS Tween for 5 min each. Finally, the membrane was detected by ECL.

#### 2.2.7. Statistical Analyses

Data presented are representatives from at least three independent experiments. Graph Pad Prism 5.0 and SPSS 23.0 were used to analyze all the data. All data were presented as mean ± SD. One-way ANOVA was used for multiple comparisons. A *P* value less than 0.05 (*P* < 0.05) was considered to be statistically significant.

## 3. Results

### 3.1. TGD Can Effectively Reduce SHR Blood Pressure

**Figure 1:**
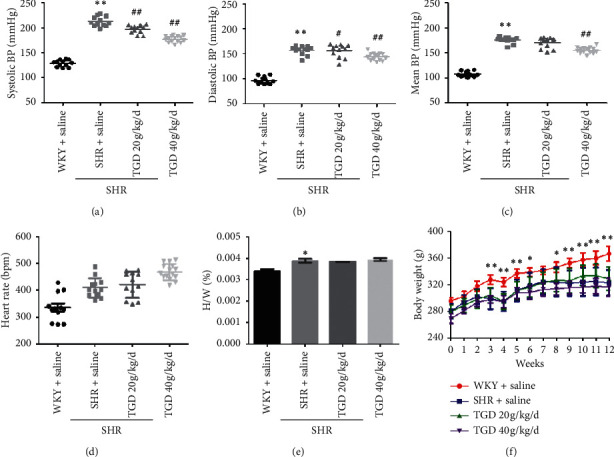
Effect of TGD on blood pressure, left ventricular wet weight, heart rate, and body weight. (a-d) 14-week-old male Wistar Kyoto (WKY) and spontaneously hypertensive rats (SHRs) were obtained. BP and heart rate were recorded for 12 weeks using a noninvasive rat tail blood pressure meter (*n* = 5 per group). (e) WKY and SHR specimens were sacrificed after dosing for 12 weeks, and the left ventricle was weighed. The bar graph displays the ratio of the left ventricular wet weight to the body weight (*n* = 5 per group). (f) Each animal's body weight was recorded weekly for 12 weeks during the experiment (*n* = 5 per group). All of the above values are shown as mean ± SD. ^*∗*^(*P*) < 0.05 compared with WKY + saline group; ^*∗∗*^(*P*) <  0.01 compared with WKY + saline group; ^#^(*P*) < 0.05 compared with SHR + saline group; ^##^(*P*) < 0.01 compared with SHR + saline group.

To evaluate the antihypertensive effect of TGD, we monitored the blood pressure of the animals for 12 weeks. The blood pressure of the SHR + saline group was significantly higher than that of the WKY group. After 12 weeks of administration of TGD, the TGD intervention groups (20 g/kg/d, 40 g/kg/d) had reduced systolic blood pressure (Figure 1(a)), diastolic blood pressure (Figure 1(b)), and mean arterial pressure (Figure 1(c)) compared with the SHR + saline group, where TGD at 40 g/kg/d was effective in controlling blood pressure after 8 weeks of administration and 20 g/kg/d was effective after 12 weeks (Figure S1). Compared with the WKY group, the left ventricle wet weight of the SHR groups was significantly increased. The left ventricle wet weight of SHR was decreased after TGD was administered, but the difference was not significant (Figure 1(f)). Compared with SHR + saline, the heart rate and weight of the TGD-treated groups did not differ (Figures 1(d) and 1(e)), indicating that TGD treatment did not affect heart rate or weight.

### 3.2. TGD Improved SHR Cardiac Function and Structure

**Figure 2:**
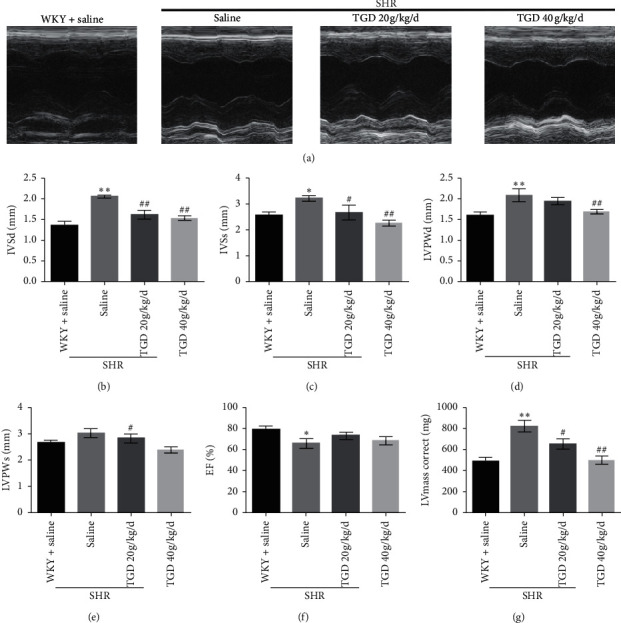
Effect of TGD on cardiac function in rats after 12 weeks of treatment. (a) Representative M-mode echocardiograms for each group. (b–g) The systolic interventricular septum (IVSs), diastolic interventricular septum (IVSd), systolic left ventricular posterior wall (LVPWs), diastolic left ventricular posterior wall (LVPWd), ejection fraction (EF%), and left ventricular mass (LVmass) of each rat were measured by echocardiography three times, and the average was taken for statistical analysis (*n* = 5 per group). Data are shown as mean ± SD. ^*∗*^(*P*) < 0.05 compared with WKY + saline group; ^*∗∗*^(*P*) < 0.01 compared with WKY + saline group; ^#^(*P*) < 0.05 compared with SHR + saline group; ^##^(*P*) < 0.01 compared with SHR + saline group.

The effects of TGD on cardiac function and structure were divulged by echocardiography. As shown by the M-mode echocardiograms (Figure 2), the left ventricular structure and function decreased in the SHR + saline group. Compared with the SHR + saline group, the ventricular structure and contraction of the TGD groups were improved (Figure 2(a)). The IVSd, IVSs, LVWPd, LVPWs, and LVmass of the SHR + TGD 40 g/kg/d group were all reduced compared with the SHR + saline group. The SHR + TGD 20 g/kg/d group also improved the above indicators, but there was no significant difference in LVPWd or LVPWs (Figures 2(b)–2(d) and 2(g)). Although the ejection fractions of the treatment groups were reduced, the improvement was not significant in either group (Figure 2(f)).

**Figure 3:**
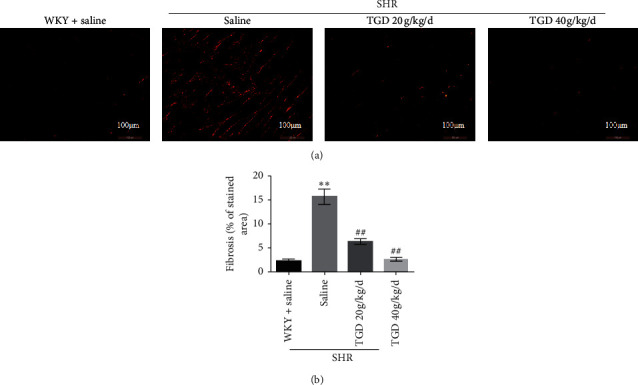
Effect of TGD on SHR cardiac fibrosis after 12 weeks of treatment. (a) Representative image of left ventricle stained with sirius red from the indicated group of rats, magnification 40 × (bottom, scale bar = 100 *μ*m). Collagen deposits show strong birefringence under polarized light microscopy. (b) Quantitative analysis of collagen content, expressed as the area of collagen in a fixed area. Data are shown as mean ± SD. ^*∗*^(*P*) < 0.05 compared with WKY + saline group; ^*∗∗*^(*P*) < 0.01 compared with WKY + saline group; ^#^(*P*) < 0.05 compared with SHR + saline group; ^##^(*P*) < 0.01 compared with SHR + saline group.

To further evaluate ventricular remodeling, we stained the rat heart with sirius red. Compared with the WKY + saline group, the left ventricle of the SHR + saline group had obvious collagen fiber deposition, suggesting a significant increase in cardiac interstitial fibrosis. After TGD treatment, cardiac interstitial fibrosis was significantly reduced in both SHR groups (Figure 3).

### 3.3. TGD Reversed Vascular Remodeling in SHR

**Figure 4:**
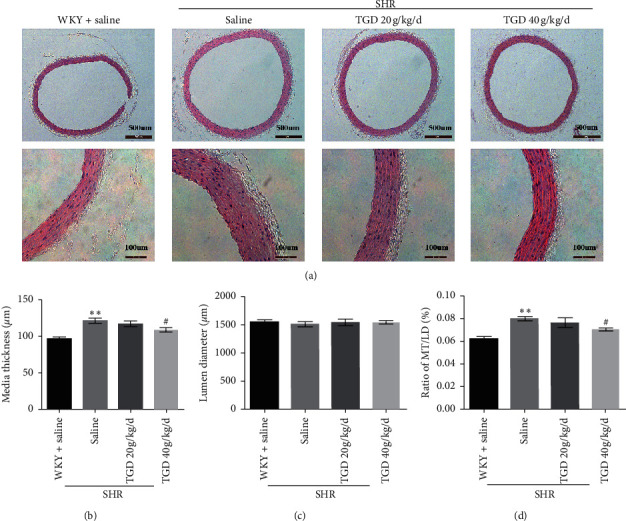
Effect of TGD treatment on aortic remodeling in SHR. (a) Histopathological changes of the thoracic aorta were observable after Harris hematoxylin and eosin (H&E) staining. Images are representatives taken at a magnification of 20 × (top, scale bar = 500um) or 40 × (bottom, scale bar = 100 um) (*n* = 3 per group); (b) media thickness (MT); (c) lumen diameter (LD); and (d) MT/LD were measured. Data are shown as mean ± SD. ^*∗*^(*P*) < 0.05 compared with WKY + saline group; ^*∗∗*^(*P*) < 0.01 compared with WKY + saline group; ^#^(*P*) < 0.05 compared with SHR + saline group; ^##^(*P*) < 0.01 compared with SHR + saline group.

The histological changes of the thoracic aorta were elucidated by Harris hematoxylin and eosin (H&E) staining. As shown in Figure 4, although there was no significant difference in lumen diameter (LD) between the three groups, the media thickness (MT) of the thoracic aorta in the SHR-control group was significantly greater than that of the WKY group, suggesting that vascular remodeling had occurred in the rats of the SHR-control group. However, thoracic aorta thickening in SHRs was significantly ameliorated by TGD treatment.

### 3.4. TGD Promoted Vasodilation and Lowered Vasoconstriction in SHR

**Figure 5:**
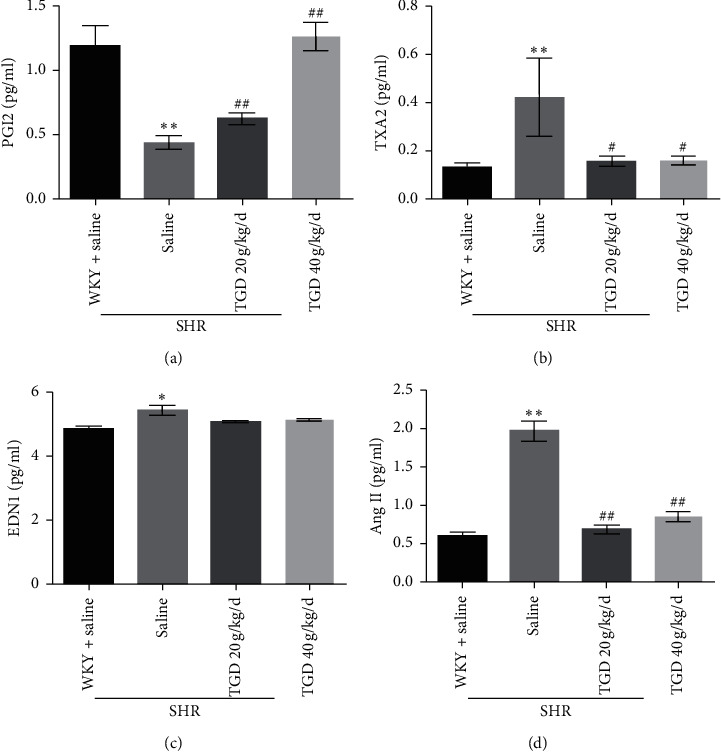
Effect of 12 weeks of TGD treatment on serum PGI2, TXA2, EDN1, and Ang II in rats. (a–d) After 12 weeks of treatment with TGD, the levels of PGI2, TXA2, EDN1, and Ang II in the serum were measured using an ELISA kit (*n* = 5 per group). Values are shown as mean ± SD. ^*∗*^(*P*) < 0.05 compared with WKY + saline group; ^*∗∗*^(*P*) < 0.01 compared with WKY + saline group; ^#^(*P*) < 0.05 compared with SHR + saline group; ^##^(*P*) < 0.01 compared with SHR + saline group.

To further determine if TGD could cause changes in vasoconstriction and diastolic factors, we performed a serum ELISA assay after 12 weeks of administration of TGD. Compared with the WKY + saline group, the relaxation factor PGI2 of the SHR + saline group was significantly reduced, while the contraction factors TXA2, EDN1, and Ang II were significantly increased. Our results showed that TGD increases the expression of vasodilating factor PGI2, compared with the SHR + saline group; TGD 40 g/kg/d has a significant difference, *P* < 0.01 (Figure 5(a)), thus supporting the notion that vasodilation decreases high blood pressure. Compared with the SHR + saline group, the TGD 20 g/kg/d, 40 g/kg/d groups had significant differences (*P* < 0.01), which reduced the expression of vasoconstrictors TXA2 and Ang II (Figures 5(b) and 5(d)), antagonizing the vasoconstriction of SHR caused by hypertension. Expression of the other vasoconstrictor, EDN1, was also reduced but not significantly in either TGD group (Figure 5(c)).

### 3.5. TGD Upregulated OPG and TRAIL in Parallel and Inhibited Apoptosis to Exert Cardiovascular Protection in SHR

**Figure 6:**
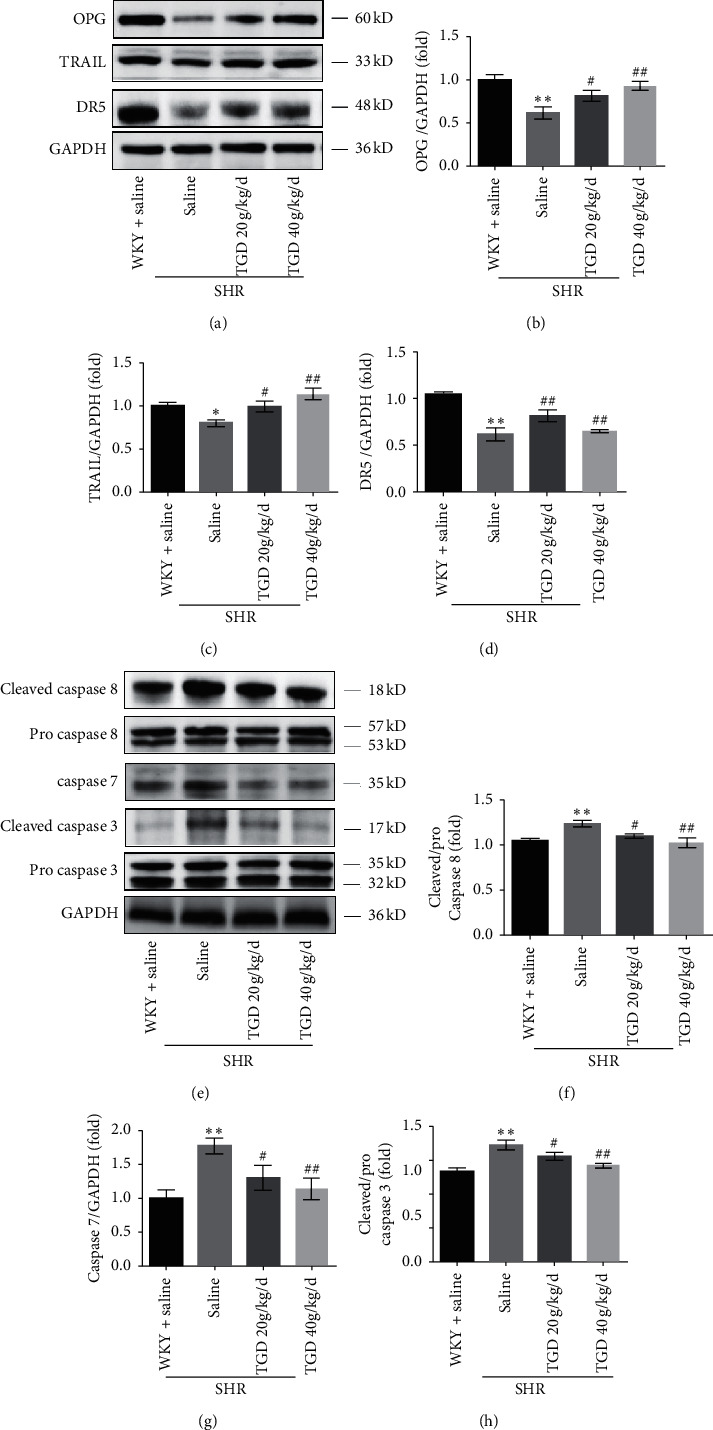
Effect of TGD on OPG, TRAIL, and the subsequent apoptotic pathway. (a–d) Representative western blots for OPG, TRAIL, DR5, GAPDH as a loading control and corresponding quantitative data (*n* = 3 per group). (e–h) Representative western blots for cleaved caspase 8, procaspase 8, caspase 7, cleaved caspase 3, procaspase 3, and GAPDH and corresponding quantitative data (*n* = 3 per group). Data are presented as means ± SD, ^*∗*^(*P*) < 0.05 compared with WKY + saline group; ^*∗∗*^(*P*) < 0.01 compared with WKY + saline group; ^#^(*P*) < 0.05 compared with SHR + saline group; ^##^(*P*) < 0.01 compared with SHR + saline group.

Earlier in our study we provided evidence that TGD ingestion is correlated with a reduction in blood pressure and a protective effect on the heart. To investigate whether the mechanism by which TGD exerts cardiovascular protection is through the interaction of OPG and TRAIL, we performed relevant analyses and found that RANKL does not seem to play a role in this system in SHR (Figure S2). In Figure 6, our research shows that the protein expression of OPG and TRAIL in the SHR + saline group was significantly reduced, and the caspase cascade was activated. After 12 weeks of TGD intervention, the expression of OPG, TRAIL, and DR5 protein in SHR was upregulated (Figures 6(a)–6(d)). At the same time, we tested the apoptotic pathway closely related to TRAIL and found that, compared with the SHR + saline group, TGD inhibited the expression of caspase 8, caspase 7, and caspase 3 in that pathway (Figures 6(e)–6(h)).

### 3.6. TGD May Inhibit Apoptosis by Upregulating AKT in SHR

**Figure 7:**
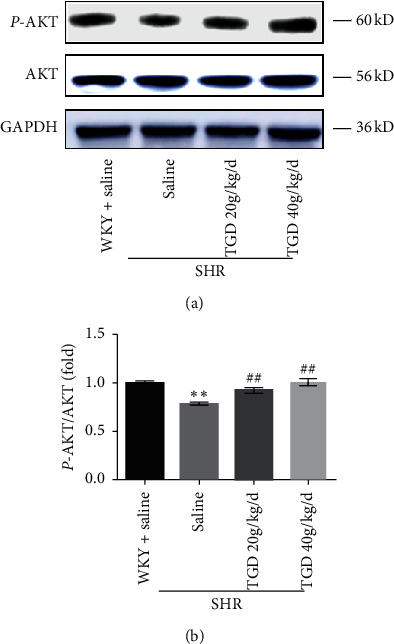
Effect of TGD on AKT protein expression in SHR. (a, b) Representative western blots for p-AKT, AKT, and GAPDH, and corresponding quantitative data (*n* = 3 per group). Data are presented as means ± SD;^*∗*^(*P*) < 0.05 compared with WKY + saline group; ^*∗∗*^(*P*) < 0.01 compared with WKY + saline group; ^#^(*P*) < 0.05 compared with SHR + saline group; ^##^(*P*) < 0.01 compared with SHR + saline group.

TRAIL can act in a manner that is proapoptotic or procell survival. Our research shows that it promotes cell survival in SHR. Therefore, we hypothesize that there may also be other ways to promote cell survival. To this end, we verified the AKT protein related to TRAIL's promotion of cell survival and proliferation based on previous studies [17]. As shown in our study, TGD can upregulate the expression of the p-AKT protein, which is consistent with the previous results to some extent (Figure 7).

## 4. Discussion

Natural products, including traditional Chinese medicine, have long been used as important treatments for various diseases in many countries around the world. Tianma Gouteng Decoction (TGD) is widely used as a common prescription for headaches and dizziness in Chinese medicine. TGD has also been widely used in Chinese hospitals and clinics to treat symptoms related to hypertension. Compared with conventional antihypertensive drugs, TGD may be an effective alternative method for treating hypertension with fewer side effects [14]. However, even when TGD has been found to be effective—as in this study—the mechanisms by which it performs its functions remain unclear. This study aimed to evaluate whether TGD resists hypertension by exerting cardiovascular protection via the OPG/TRAIL system.

The main finding of this study was that TGD lowered the blood pressure in SHR, especially after 8 weeks of administration. TGD at 40 mg/kg/d significantly reduced systolic, diastolic, and mean arterial pressure (Figure S1). Furthermore, there was no significant difference in body weight and heart rate between the SHR + TGD groups and the SHR + saline group. This implies that TGD does not cause toxic effects such as weight loss and heart rate changes. As can be seen from Figure 1, the antihypertensive effect of TGD is tempered and smooth, which may avoid the known adverse effects of some antihypertensive drugs [18]. In addition, the dual effects of direct vasodilation and long-term vascular reverse remodeling of TGD support the effectiveness of multitarget strategies in blood pressure control, which may be an advantage of traditional Chinese medicines with multiple components.

To study the cardiovascular protective mechanism of TGD, we evaluated the changes in cardiac function and structure, vascular remodeling, and changes in vasoconstriction and relaxation factors during the hypertension. Echocardiography demonstrates that TGD can reverse left ventricular remodeling and reduce heart weight in SHR but has no significant effect on EF. It can also improve cardiac fibrosis. In addition, HE staining showed that TGD treatment significantly reduced the thickening of the thoracic aorta in the SHR model, indicating that it reversed the effect of vascular remodeling. Therefore, we believe that TGD can exert cardiovascular protection by inhibiting heart and vascular remodeling, which are closely related to the reduction of blood pressure. Correspondingly, our results show that TGD treatment can significantly reduce the contractile factors TXA2, Ang II, and EDN1, which cause an increase in blood pressure in the SHR model and increase the expression of PGI2.

The vascular calcification (VC) process can generally be described as progressive arteriosclerosis that results in reduced vascular elasticity leading to hypertension or atherosclerosis, ultimately increasing the risk of cardiovascular events [19]. OPG is a soluble member of the tumor necrosis receptor superfamily, which exerts its biological function by acting as a decoy receptor for RANKL or TRAIL. A preponderance of evidence shows that VC may involve OPG/RANKL/TRAIL, a signaling pathway traditionally associated with bone remodeling. The receptor activator of NF-*κ*B ligand (RANKL) promotes VC, while osteoprotegerin (OPG) acts as a RANKL decoy receptor to block this effect, which is contrary to the known functional effects of these proteins in bone metabolism. However, in clinical studies, high levels of plasma OPG have been proven to positively predict the morbidity and mortality of CVD. Therefore, OPG plays a dual role in the cardiovascular system [20]. In addition, evidence suggests that tumor necrosis factor-related apoptosis-inducing ligand (TRAIL) is another bait ligand for OPG that may produce calcification in the vasculature [21]. But TRAIL also has a dual role. There is evidence that TRAIL induces apoptosis in vitro by induction of ECs [22] and VSMC [23], but interestingly, under certain conditions, TRAIL has also been shown to have antiapoptotic activity in these cells. Study has shown that it can promote the survival and proliferation of primary human vascular endothelial cells by activating the AKT pathway [13], and studies have also showed that administration in the form of recombinant soluble TRAIL or AAV-TRAIL expression viral vectors reduces the development of cardiomyopathy in a ApoE-/-diabetic mouse model and shows potential cardioprotective activity [24]. This is consistent with our research results. In general, OPG exhibits an opposite effect on the skeletal system as it does on vasculature, that is, protecting the blood vessels, while TRAIL has a substantial but diverse functional role in the vasculature, both dependent on and independent of OPG. Our study shows that TGD can significantly increase the expression of OPG and TRAIL, which rise in parallel. At the same time, it increased the expression of p-AKT protein and decreased caspase cascade, thereby playing a cardiovascular protective effect (Figures 5 and 6). This may be related to the ability of TRAIL to inhibit the apoptosis process of vascular endothelia [25], which may be partly independent of OPG. However, the western blot analysis of OPG's other ligand RANKL and the calculation of the ratio of OPG to TRAIL did not show any significant difference (Figure S2). The antiapoptotic effect in the cardiovascular protection of hypertension may be partly independent of OPG, but there is no definite evidence. It was also verified that TRAIL may inhibit inflammation through apoptotic-independent pathways, by up-=regulating AKT to promote cardiomyocyte survival and thus inhibit apoptosis [13].

In conclusion, our study indicates that TGD can increase the protein expression of OPG and TRAIL, activate p-AKT, and inhibit the caspase cascade, thereby inhibiting cardiomyocyte apoptosis and exerting cardiovascular protection. However, because TGD is composed of a variety of Chinese herbal medicines, it contains many compounds. The number of antihypertensive active ingredients in these compounds is unknown, and the signaling pathways through which they function are also unknown. Thus, in the course of developing better drugs for the treatment of hypertension, these important questions should be addressed through future research.

## Data Availability

The data supporting the findings of this study are available from the corresponding author upon request.

## Supplementary Materials

Supplementary Materials Figure S1: effect of TGD on blood pressure. Figure S2: effect of TGD on RANKL, and OPG/TRAIL ratio.
