# Supplementary material for: Tianma Gouteng Decoction Exerts Cardiovascular Protection by Upregulating OPG and TRAIL in Spontaneously Hypertensive Rats
Source: Evid Based Complement Alternat Med. 2020 Oct 20;2020:3439191. doi: 10.1155/2020/3439191 (PMC7593748; doi:10.1155/2020/3439191)
Supplement: Supplementary Materials — Figure S1: effect of TGD on blood pressure. Figure S2: effect of TGD on RANKL, and OPG/TRAIL ratio. [file 3439191.f1.pdf]

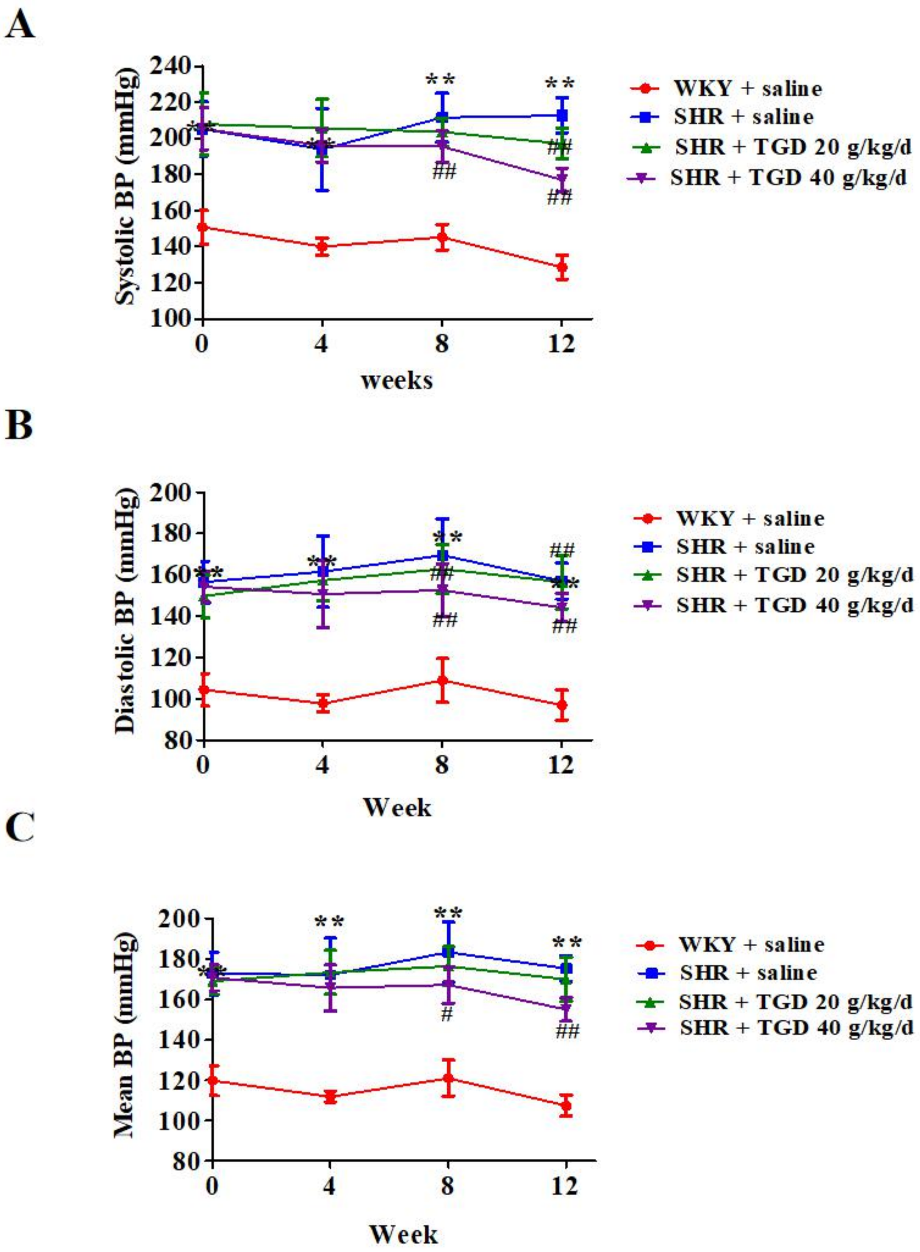

Figure S1. Effect of TGD on blood pressure. (A-C) 14 week-old male Wistar-Kyoto (WKY) and spontaneously hypertensive rats (SHRs) were obtained. BP and heart rate were recorded for 12 weeks using a non-invasive rat tail blood pressure meter (n=5 per group). All of the above values are shown as mean ± SD. * *P* < 0.05 compared with WKY + saline group; ** *P* < 0.01 compared with WKY + saline group; # *P* < 0.05 compared with SHR + saline group, ## *P* <0.01 compared with SHR + saline group.

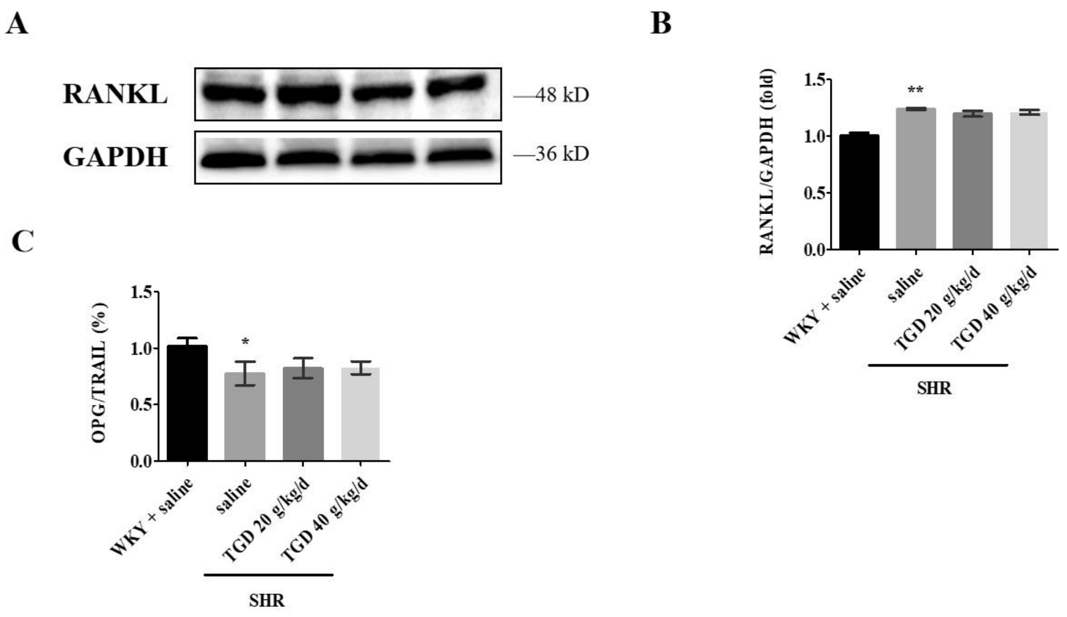

Figure S2. Effect of TGD on RANKL, and OPG/TRAIL ratio. (A, B) Representative digital western blots for RANKL and GAPDH, and corresponding quantitative data (n=3 per group). (C) OPG/TRAIL was measured. All of the above values are shown as mean ± SD. * $P < 0.05$ compared with WKY + Saline group; ** $P < 0.01$ compared with WKY + Saline group; # $P < 0.05$ compared with SHR + Saline group, ## $P < 0.01$ compared with SHR + Saline group.
